# The first discovery of *Polypedatesteraiensis* (Dubois, 1987) (Rhacophoridae, Anura) in China

**DOI:** 10.3897/BDJ.12.e127029

**Published:** 2024-07-05

**Authors:** Zhong-Bin Yu, Felista Kasyoka Kilunda, Kai Wang, Yu-Yang Cao, Chun-Lian Wu, Zheng-Pan Duan, Chang-Sheng Zuo, Ding-Can Zhang, Yun-He Wu, Jing Che

**Affiliations:** 1 Key Laboratory of Genetic Evolution and Animal Models, and Yunnan Key Laboratory of Biodiversity and Ecological Conservation of Gaoligong Mountain, Kunming Institute of Zoology, Chinese Academy of Sciences, 650223, Kunming, Yunnan, China Key Laboratory of Genetic Evolution and Animal Models, and Yunnan Key Laboratory of Biodiversity and Ecological Conservation of Gaoligong Mountain, Kunming Institute of Zoology, Chinese Academy of Sciences, 650223 Kunming, Yunnan China; 2 Southeast Asia Biodiversity Research Institute, Chinese Academy of Sciences, 05282, Yezin, Nay Pyi Taw, Myanmar Southeast Asia Biodiversity Research Institute, Chinese Academy of Sciences, 05282 Yezin, Nay Pyi Taw Myanmar; 3 Kunming College of Life Science, University of the Chinese Academy of Sciences, 650204, Kunming, Yunnan, China Kunming College of Life Science, University of the Chinese Academy of Sciences, 650204 Kunming, Yunnan China; 4 Key Laboratory of Southwest China Wildlife Resources Conservation, China West Normal University, Ministry of Education, 637009, Nanchong, Sichuan, China Key Laboratory of Southwest China Wildlife Resources Conservation, China West Normal University, Ministry of Education, 637009 Nanchong, Sichuan China; 5 Administrative Bureau of Tongbiguan Provincial Nature Reserve, 679300, Dehong, Yunnan, China Administrative Bureau of Tongbiguan Provincial Nature Reserve, 679300 Dehong, Yunnan China

**Keywords:** *Polypedatesteraiensis*, new record, Gaoligong Mountain, China

## Abstract

**Background:**

The genus of *Polypedates* Tschudi, 1838 currently comprises 25 recognised species with four of these species reported in Yunnan, China. Dubois (1987) speculated the distribution of *P.teraiensis* in China; however, there was no study carried out to confirm its distribution in the region.

**New information:**

We herein describe *P.teraiensis* as a new national record, based on a specimen collected from Yunnan border region. Phylogenetically, our sequence clustered with the sequences of recognised *P.teraiensis* specimens from Bangladesh, Myanmar and India. The uncorrected pairwise distances between the specimens from China and other *P.teraiensis* localities was small, ranging from 0.0-0.7%, based on 16S rRNA gene. Therefore, we report *P.teraiensis* as a new species record for China.

## Introduction

Whipping Frogs of the genus *Polypedates* Tschudi, 1838 are widely distributed across eastern India, south-eastern Himalayas, southern China, the Indochina Peninsula and various Southeast Asian islands. To date, the genus consists of 25 recognised species, with four species known to occur in China, including *P.braueri* (Vogt, 1911), *P.impresus* Yang, 2008, *P.megacephalus* Hallowell, 1861 and *P.mutus* (Smith, 1940) (AmphibiaChina 2024, Frost 2024). In addition to the above-mentioned four species, another species, *P.teraiensis* (Dubois, 1987) was speculated to occur in China as well, as the species is known to occur in nearby countries such as Nepal, India, Bangladesh and Myanmar (e.g. Ao et al. (2003), Daniels (2005), Ahmed et al. (2009), Tshewang and Letro (2018), Hakim et al. (2020), Purkayastha et al. (2020), Rabbe et al. (2022), Raj et al. (2023)). However, such speculation has not been validated by voucher specimens to date.

During our field investigation of amphibians and reptiles of the Gaoligong Mountain in 2023, we collected a single specimen from Yingjiang County in south-western Yunnan Province, China, close to the China-Myanmar-border. Based on phylogenetic analysis, we identified it as conspecific with *P.teraiensis*, which represents the first confirmed voucher for the speculated distribution of the species in China. Here, we confirm the distribution of *P.teraiensis* in China and provide a description of the Chinese specimen.

## Materials and methods

**Figure 1.**
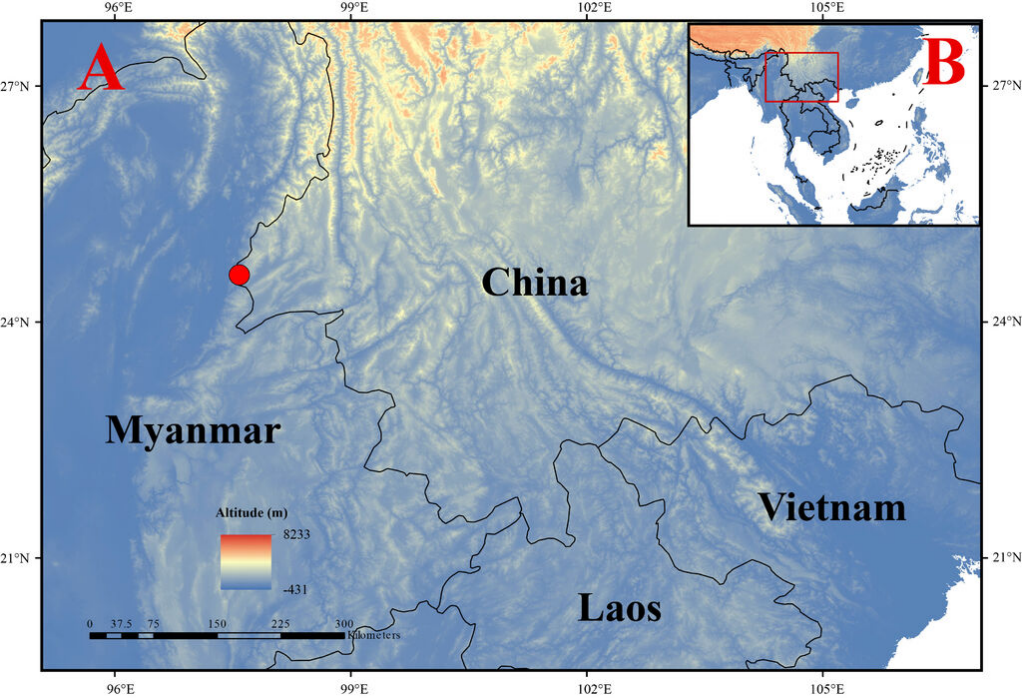
Map showing the location of the new record of *P.teraiensis* in China (red circle).

The field survey was conducted in the Tongbiguan Provincial Nature Reserve under the permit issued by the Dehong Prefecture Forestry and Grassland Bureau of Yunnan Province (Fig. 1). The specimen was photographed, then euthanised and fixed in 75% ethanol for permanent storage. A liver tissue sample was collected and preserved in absolute ethyl alcohol for molecular analysis. The specimen was deposited at the Kunming Natural History Museum of Zoology, Kunming Institute of Zoology, Chinese Academy of Sciences (KIZ).

Morphological measurements were taken using a digital caliper to the nearest 0.1 mm (Suppl. material 2). Morphological terminology followed Fei et al. (2009). Measurements included 17 morphological characteristics: (1) Snout-vent length (SVL); (2) Head length (HL); (3) Head width (HW); (4) Snout length (SL); (5) Distance from the centre of the nostril to the tip of the snout (SN); (6) Nostril-eye distance (N-EL); (7) Eye diameter (ED); (8) Tympanum diameter (TD); (9) Internarial distance (IND); (10) Interorbital distance (IOD); (11) Upper eyelid width (UEW); (12) Length of lower arm and hand (LAHL); (13) Hand length (HAL); (14) Hind-limb length (HLL); (15) Thigh length (THL); (16) Tibia length (TL) and (17) Foot length (FL).

Genomic DNA was extracted from the liver tissue using the standard phenol-chloroform extraction protocol (Sambrook et al. 1989). Partial fragments of the mitochondrial 16S rRNA were amplified and sequenced for this sample using the primer pairs (5’-3’) 16S rRNA-F (CGCCTGTTTAYCAAAAACAT) and 16S rRNA-R (CCGGTYTGAACTCAGATCAYGT) (Kocher et al. 1989). The polymerase chain reaction (PCR) was performed in a 25 μl reaction volume with the following cycling conditions: initial denaturation step at 95°C for 5 min, 35 cycles of denaturation at 95°C for 1 min, annealing at 55°C for 1 min, extension at 72°C for 1 min and final extension at 72°C for 10 min. The products were purified and sequenced by Tsingke Biotechnology (Beijing) Co., Ltd., using the same primers employed in the PCR process in both forward and reverse directions. Sequencing was performed using the BigDye Terminator Cycle Sequencing Kit on an ABI PRISM 3730 DNA Analyzer (Applied Biosystems, Foster City, CA, USA). The newly-obtained nucleotide sequence was first assembled and edited using DNASTAR LASERGENE 7.1. after which the sequence was deposited in the GenBank.

Phylogenetic relationships within the genus *Polypedates* were inferred from 16S rRNA. The homologous sequences of the genus *Polypedates* and the outgroup species (*Chirixalusnongkhorensis*, *Zhangixalusdennysi* and *Rhacophorusnorhayatii*), were downloaded from GenBank (Clark et al. 2016) (Suppl. material 1). Sequences were aligned using MUSCLE 3.8 (Edgar 2004), then checked by eye for accuracy and trimmed to minimise missing characters in MEGA 6.0.6 (Tamura et al. 2013).

Phylogenetic reconstruction was performed using Bayesian Inference (BI) and Maximum Likelihood (ML) methods, based on the 16S rRNA gene. The best-fit substitution model of evolution was selected under the Bayesian Information Criterion (BIC; Posada (2008)) by the programme jModelTest 2.1.7 (Darriba et al. 2012). BI analysis was implemented by the CIPRES web server (Miller et al. 2010). The BI analyses were conducted with 10 million generations using the SYM+G model and sampled every 1000 generations. Convergence was assessed in Tracer 1.5 (Rambaut and Drummond 2009), based on having an average standard deviation of split frequencies less than 0.01 and ESS values great than 200. We excluded the first 25% of trees as burn-in before the log-likelihood scores stabilised. Maximum Likelihood analyses were performed using RAxML-HPC BlackBox 8.2.10 (Stamatakis 2014) on the CIPRES Science Gateway (Miller et al. 2010). The analyses used the proportion of invariable sites estimated from the data and 1,000 bootstrap pseudoreplicates under the GTR+gamma model.

## Data resources

**Figure 2.**
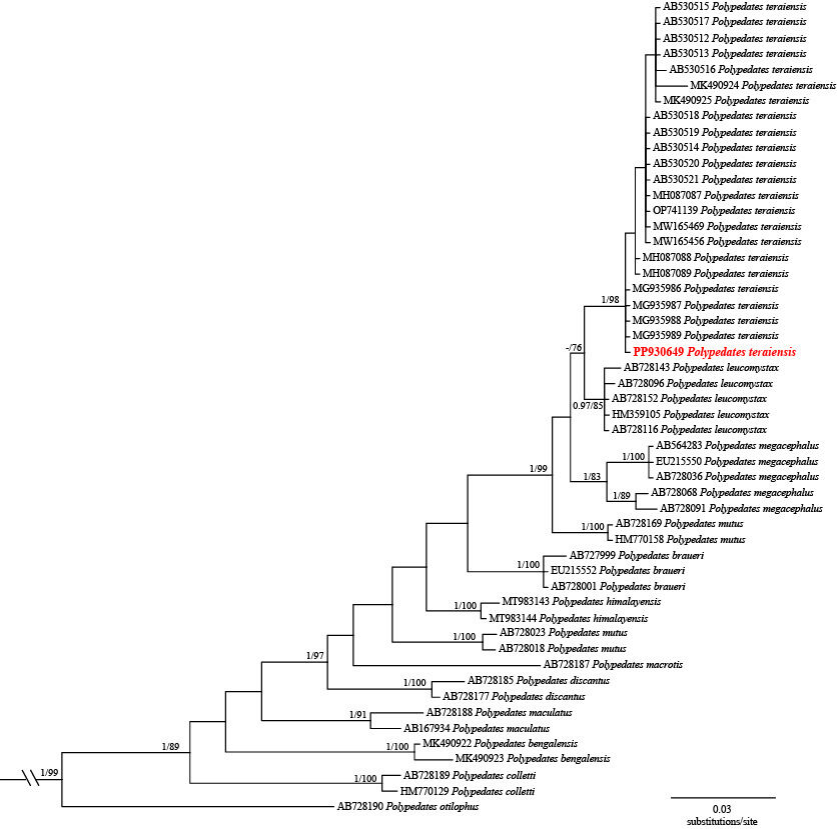
Bayesian Inference (BI) and Maximum Likelihood (ML) analysis of the genus *Polypedates* from partial DNA sequences of the mitochondrial 16S rRNA gene. Nodal support values with Bayesian posterior probabilities (BPP) > 0.95/ML inferences (BS) > 70 were performed near the respective nodes. “-” represents Bayesian posterior probability < 0.95 and bootstrap support < 70. Bayesian posterior probabilities (BPP) < 0.95/ML inferences (BS) < 70 are not shown.

The aligned 16S rRNA dataset contained a total of 507 nucleotide base pairs (bp), with 165 variable positions and 126 parsimony informative sites (including outgroups). The ML and BI trees had essentially identical topologies and most terminal clades obtained relatively high support values, except for some internal nodes (Fig. 2). The male specimen collected from Tongbiguan Township, Yingjiang County, Yunnan, China, clustered with the specimens of *P.teraiensis* from Myanmar, India and Bangladesh with strong support (Bayesian posterior probability (BPP) = 1.00, bootstrap support (BS) = 98) for both Bayesian Inference and Maximum Likelihood analysis (Fig. 2). The genetic distance (uncorrected *p*-distance) between the specimen from China and the specimens of *P.teraiensis* from other regions was found to be very small (0.0-0.7%, Suppl. material 3). According to phylogenetic data, we confirm the specimen from Tongbiguan Nature Reserve as *P.teraiensis* and we provide a detailed description of the Chinese specimen.

## Taxon treatments

### Polypedates teraiensis

(Dubois, 1987)

73CFF8CA-48DF-5F88-A508-87D1B2942CCF

#### Materials

- **Type status:** Other material. **Occurrence:** catalogNumber: KIZ 051716; individualCount: 1; sex: male; lifeStage: adult; occurrenceID: D4B5A174-7CF0-5725-A51B-1522939853AD; **Taxon:** scientificNameID: *Polypedatesteraiensis*; class: Amphibia; order: Anura; family: Rhacophoridae; genus: Polypedates; specificEpithet: *teraiensis*; **Location:** continent: Asia; country: Yingjiang; countryCode: CHN; stateProvince: Yunnan; county: China; locality: Tongbiguan; verbatimElevation: 1167 m; verbatimLatitude: 24°35′52.20″; verbatimLongitude: 97°34′51.96″; **Event:** year: 2023; **Record Level:** institutionCode: KIZ

#### Description

**Figure 3.**
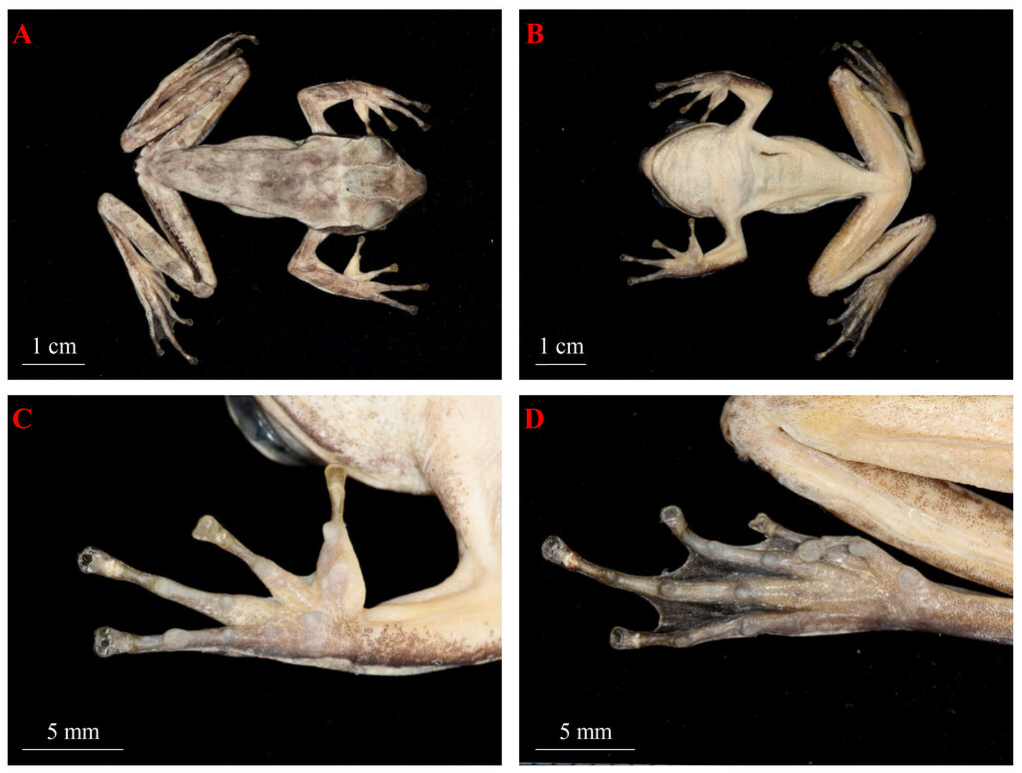
Male of *Polypedatesteraiensis* (KIZ 051716) in preservative. **A** Dorsal view; **B** Ventral view; **C** Ventral view of finger; **D** Ventral view of toe.

Adult medium-sized male, body flat (SVL 47.8mm); head moderate larger than one-third of snout-vent length (HL/SVL 0.36); head length slightly larger than width (HW/HL 93.0%); snout blunt, round, obtuse beyond lower jaw in ventral view; snout length slightly less than half of head length (SL/HL 46.8%); dorsal head slightly concave; canthus rostralis distinct; loreal region slightly concave, near vertical; nostril oval, closer to snout than eyes (SN/N-EL 43.4%); internarial distance less than interorbital distance (IND/IOD 79.2%), but larger than upper eyelid width (IND/UEW 105.0%); eyes large, about one-third of head length (ED/HL 32.7%); tympanum distinct, oval, larger than two-third of eye diameter (TD/ED 69.6%); supratympanic fold distinct, slender, extend from posterior eye to above shoulder; maxillary teeth single row, small; tongue heart-shaped, deeply notched posteriorly, posterior 1/3 free; vomerine teeth two short rows, prominently, untouching inner front edges of choanae, separated by distance less than length of each series; male with internal subgular vocal sacs, vocal sac opening on floor of mouth at each corner (Fig. 3).

Fore-limbs robust; lower arm and hand length slightly less than half of SVL (LAHL/SVL 48.7%), hand length less than one-third of snout-vent length (HAL /SVL 29.9%); fingers slender, dorsally compressed, webbing free, relative length of the fingers: III > IV > II ≈ I; fringe present, weakly developed; fingers tips nearly rounded, dilated into large disc distally, circummarginal grooves present; subarticular tubercles prominent, rounded, formula 1, 1, 2, 2; supernumerary tubercle absent; metacarpal tubercles three: inner metacarpal largest, oblong elliptical; outer metacarpal smallest, rounded; middle one moderate, oval; nuptial pad present on dorsal base of first finger (Fig. 3).

Hind-limbs slender, long (HLL/SVL 158.8%); tibia length slightly longer than thigh length (TL/THL 97.9%), much longer than foot length (FL/SHL 83.1%), slightly longer than half of SVL; tibia-tarsal articulation reaching beyond anterior border of eye when hind-limbs are stretched alongside body; heels overlap when legs held at right angles to body; relative length of toes IV > III > V > II > I; toe tips dilated into large disc with circummarginal grooves, nearly rounded, smaller than discs on fingers; toes half-webbed; subarticular tubercles distinct, round, formula: 1, 1, 2, 3, 2; supernumerary tubercle absent; inner metatarsal tubercle present, oblong; outer metatarsal tubercle absent; no tarsal fold (Fig. 3).

Dorsal skin relatively smooth with small granular, dorsolateral fold absent; throat smooth with small tubercles, indiscernible; chest, belly and ventral thigh speckled with small tubercles (Fig. 3).

**Colouration in preservative**

Dorsal surface is faded to greyish-brown, mottled with dark patches; transverse bars on the back of the limbs; black stripes under the temporal fold; ventral surface creamy white, with brown pigment particles on the chest.

#### Distribution

Our study further extends the species' distribution range to Yunnan, China. Therefore, the species currently known from the Dehong Prefecture, south Yunnan, China, eastern Nepal, Bhutan, India, Bangladesh and Myanmar.

#### Ecology

This species often inhabits evergreen broad-leaved forests. We found it in the shrubbery by the stream during summer nights. This species is in sympatric distribution with *Amolopsafghanus* and *Zhangixalussmaragdinus*.

#### Notes

**Chinese Names** We suggest “Nán Yà Fàn Shù Wā (南亚泛树蛙)” as its Chinese common name.

## Discussion

Yunnan has a rich species diversity and is often referred to as the "kingdom of animals". Within it lies the Gaoligong Mountain situated in the border region between western Yunnan, China and Myanmar, at the intersection of three biodiversity hotspots (the Himalayas, Indo-Burma and the mountains of southwest China) (Myers et al. 2000, Mittermeier et al. 2004). This region boasts a rich species diversity, especially the Yingjiang County located in the southern part of the Gaoligong Mountain (Li et al. 2024, Wu et al. 2024). Over the years, several new species and new species records have been continuously discovered in this region, an indicator that the area’s biodiversity may have been greatly underestimated, thus underscoring the need for further exploration and investigation efforts (e.g. Yang and Chan 2018, Yang et al. 2018, Wu et al. 2021, Yu et al. 2022, Zhang et al. 2022, Wu et al. 2023, Wu et al. 2024). Following our field surveys of the Gaoligong Mountain, we recorded three species of *Polypedates*. Our study increased the known species number of *Polypedates* in China to five, further supporting the conclusion that amphibian diversity in the Gaoligong Mountain still remains underestimated.

Our field survey also revealed that three species of the genus *Polypedates* are sympatric: *P.braueri*, *P.impresus* and *P.teraiensis*. These three sympatric species from the Tongbiguan Provincial Nature Reserve, Yunnan Province, are difficult to distinguish from each other, solely based on morphological evidence. The application of molecular methods is crucial for reliable identification as it guides morphological re-examinations, further elucidating fine-scale differences in morphological characteristics that represent species-specific variations. Moreover, our understanding of the mechanisms driving sympatric speciation in these species remains limited. Future studies that integrate additional data, such as acoustic evidence and genomics, hold promise in addressing this question comprehensively.

## Supplementary Material

XML Treatment for Polypedates teraiensis

75DB5BDF-060E-5902-ACE5-ACA68834B64F 10.3897/BDJ.12.e127029.suppl1 Supplementary material 1 Table S1 Data type Sampling information Brief description Table S1. Localities, voucher ID and GenBank numbers for all samples used in this study. File: oo_1074570.xlsx https://binary.pensoft.net/file/1074570 Zhong-Bin Yu, Felista Kasyoka Kilunda, Kai Wang, Yu-Yang Cao, Chun-Lian Wu, Zheng-Pan Duan, Chang-Sheng Zuo, Ding-Can Zhang, Yun-He Wu, Jing Che

CFA8B99F-5F39-5835-B7D3-A751285D38ED 10.3897/BDJ.12.e127029.suppl2 Supplementary material 2 Table S2 Data type Morphological data Brief description Measurement (in mm) and proportions of the *Polypedatesteraiensis*. File: oo_1041598.docx https://binary.pensoft.net/file/1041598 Zhong-Bin Yu, Felista Kasyoka Kilunda, Kai Wang, Yu-Yang Cao, Chun-Lian Wu, Zheng-Pan Duan, Chang-Sheng Zuo, Ding-Can Zhang, Yun-He Wu, Jing Che

557EED7D-DFB0-528E-9557-70232A70F1F9 10.3897/BDJ.12.e127029.suppl3 Supplementary material 3 Table S3 Data type Average uncorrected p-distances Brief description Average uncorrected p-distances amongst the Polypedates individuals calculated from 16S rRNA gene sequences. File: oo_1041599.xlsx https://binary.pensoft.net/file/1041599 Zhong-Bin Yu, Felista Kasyoka Kilunda, Kai Wang, Yu-Yang Cao, Chun-Lian Wu, Zheng-Pan Duan, Chang-Sheng Zuo, Ding-Can Zhang, Yun-He Wu, Jing Che
