# Supplementary material for: The first discovery of Polypedatesteraiensis (Dubois, 1987) (Rhacophoridae, Anura) in China
Source: Biodivers Data J. 2024 Jul 5;12:e127029. doi: 10.3897/BDJ.12.e127029 (PMC11249848; doi:10.3897/BDJ.12.e127029)
Supplement: Supplementary material 2 — Table S2 [file bdj-12-e127029-s002.docx]

**Table S2.** Measurement (in mm) of and proportions of the *Polypedates teraiensis*.

| **Characters** | **KIZ 051716** | **Ratio (-/SVL)** |
| --- | --- | --- |
| SVL | 47.8 | - |
| HL | 17.1 | 35.8% |
| HW | 15.9 | 33.3% |
| SL | 8.0 | 16.7% |
| ED | 5.6 | 11.7% |
| IOD | 5.3 | 11.1% |
| UEW | 4.0 | 8.4% |
| IND | 4.2 | 8.8% |
| N-EL | 5.3 | 11.1% |
| SN | 2.3 | 4.8% |
| TD | 3.9 | 8.2% |
| LAHL | 23.3 | 48.7% |
| HAL | 14.3 | 29.9% |
| HLL | 75.9 | 158.8% |
| THL | 23.8 | 49.8% |
| TL | 24.3 | 50.8% |
| FL | 20.2 | 42.3% |
